# An electrospun scaffold loaded with anti-androgen receptor compound for accelerating wound healing

**DOI:** 10.4103/2321-3868.118935

**Published:** 2013-09-18

**Authors:** Cassandra Chong, Yiwei Wang, Peter K. M. Maitz, Ulla Simanainen, Zhe Li

**Affiliations:** 1 Burns & Reconstructive Surgery Research Group, Australia; 2 Andrology Group of ANZAC Research Institute, Australia; 3 Burns Unit, Concord Repatriation General Hospital, Concord, New South Wales 2139 Australia

**Keywords:** Wound healing, electrospun scafold, adrogen receptor inhibition, ASC-J9

## Abstract

Current dermal regenerative scafolds provide wound coverage, and structural support and guidance for tissue repair, but usually lack enough bio-signals needed for speeding up skin cell growth, migration, wound closure, and skin regeneration. In this study, an androgen receptor (AR) inhibitor called ASC-J9 is used to demonstrate the concept and feasibility of fabricating drug-loaded scafolds via electrospinning. Inhibition of androgen is known to promote skin wound healing. The novel ASC-J9 — loaded porous scafold was fabricated for skin wound repair using electrospun fibers of collagen and polycaprolactone (PCL) blend. Our preliminary results indicated that ASC-J9 — loaded scafolds facilitated more efficient attachment and ingrowth of dermal fbroblasts, compared to the control collagen-PCL scafold. A signifcant increase of cell proliferation was observed with the drug-loaded scafold over a 28-day period. The drug-loaded scafold also accelerated keratinocyte migration and wound closure in a contraction-inhibited mouse wound model over 21 days. The data indicated a sustained release of ASC-J9 from the scafold and its potential to accelerate wound healing by promoting cell proliferation and migration over an extended period of time. More importantly, our results proved the concept and feasibility of fabricating drug-releasing or bioactive dermal scaffolds for more efective wound healing.

## Introduction

The patients with extensive burn injuries always have limited donor sites for skin grafting. Delayed wound closure, due to the lack of autologous skin grafts and the inability of skin cell migration from neighboring healthy tissue, could result in severe wound contraction and scarring.[1–3]

**Table 1:** 

Access this article online	
**Quick Response Code:**	**Website:** www.burnstrauma.com
	**DOI:** 10.4103/2321-3868.118935

Skin tissue engineering aims to develop an alternative for skin grafts that can mimic natural skin structure, facilitate wound healing and tissue regeneration, and restore skin function. Dermal regenerative bioscaffold, stem cell resources, and various biofactors are essential for skin tissue engineering. Currently, there are many researches of skin engineering that have focused mainly on scaffold structures and morphology, especially the properties such as porosity and pore size, and how they influence cell-scaffold interactions. While current dermal regenerative templates promote wound healing and provide wound closure, they are still far from being perfect. The niche environment of current dermal scaffolds usually lacks a sufficient level of biological cues that could induce or stimulate cell migration, proliferation, and differentiation to accelerate wound healing and better skin regeneration. Consequently, delayed wound healing could lead to severe scarring and wound contraction. The unsatisfactory outcome, in fact, highlighted an aspect of skin tissue engineering that needs further improvement, which is to develop a better scaffold that could provide biological cues as well as a temporal and spatial environment for scaffolding of cells.

Deliveries of various biofactors including epidermal growth factor (EGF) and fibroblast growth factor (FGF) have been a trendy strategy for promoting angiogenesis, wound healing, and tissue engineering. Hormones and growth factors including androgen, EGF, FGF, and angiogenic factors are known to play critical roles in skin regeneration. They could stimulate the proliferation and migration of keratinocytes, fibroblasts, and endothelial cells to promote angiogenesis and wound healing.[4,5] The regulation of androgen and its receptors could modulate wound healing.[6] The hydrogel or gelatin microsphere was proved effective as matrices for controlled delivery of growth factors for wound healing.[7,8] However, to produce a growth factor-releasing electrospun scaffold remains a challenge as the growth factor could lose its bioactivity during the harsh process of scaffold fabrication.[7] In this study, we tried an alternative strategy to incorporate a chemical compound that was demonstrated to regulate the activities of androgen in wound healing. The drug chosen for this study was ASC-J9 (5-hydroxy-1,7-bis (3,4-dimethoxyphenyl)-1,4,6-heptatrien-3-one), a synthetic anti-androgen agent[8] which enhances androgen receptor (AR) degradation.[6,9] It is of the same family as flutamide that blocks the interaction between androgens and their receptors to promote wound closure.[6] ARs have been found to be expressed in the keratinocytes and dermal fibroblasts of healing skin[6] and are able to induce collagen expression in dermal fibroblasts.[10] ARs are also thought to indirectly modulate fibroblast proliferation in addition to collagen synthesis via Transforming growth factor (TGF)-β1.[11–13] A previous study showed that topical application of an ASC — J9 — containing cream accelerated re-epithelialization which was likely to be a result of suppression of local tumor necrosis factor (TNF)-α expression.[9] Due to ASC-J9’s chemical stability and advantageous results in promoting wound healing, it was chosen for this study to demonstrate the principle of drug release from electrospun scaffolds for wound healing.

The aim of this study was, therefore, to develop a bioactive scaffold which harnessed the properties of ASC-J9 to accelerate wound healing in a collagen — polycaprolactone (PCL) — ASC-J9 (CPA) composite scaffold. The bioactive scaffolds could potentially facilitate more efficient cell migration, growth, and differentiation for wound healing and skin regeneration.

## Materials and methods

### Materials

Rat tail collagen type I was purchased from BD Biosciences, San Jose, USA. PCL (Mn 80,000), 1,1,1,3,3,3-hexafluoro-2-propanol (HFP), diphosphate buffer solution (DPBS), phosphate buffer solution (PBS), Dulbecco’s modified eagle’s medium (DMEM), sodium cacodylate buffer trihydrate, penicillin/streptomycin/neomycin, and hexamethyldisilazane (HMDS) were obtained from Sigma-Aldrich, Castle Hill, Australia. Paraformaldehyde and glutaraldehyde were purchased from ProSciTech, Kirwan, Australia. Triton solution, ProLong® Gold antifade reagent with 4′,6-diamidino-2-phenylindole (DAPI), and fetal bovine serum (FBS) were obtained from Life Technology, Mulgrave, Australia. ASC-J9 was purchased from Sapphire Biosciences, Waterloo, Australia. Primary human dermal fibroblasts (HDFs) were isolated and cultured in this study following the approval of Concord Hospital Human Ethics Committee.

### Production and characterization of collagen-PCL (CP) and collagen-PCL-(ASC-J9) (CPA) scaffolds

Collagen was chosen for its well-known, cell-interactive properties and PCL for its mechanical strength and biodegradable, biocompatible, and bioabsorbable characteristics.

Polymer solutions were prepared by dissolving polymers in HFP: 7.5% (w/v) collagen and 7.5% (w/v) PCL for CP scaffolds and addition of 0.1 mg/ml ASC-J9 to the CP solution to form the drug-loaded CPA scaffolds.[9] The solution was loaded into a 1-ml syringe with a blunt 18-gauge needle attached. Flow rate was regulated at 3 ml/h and dispensed volume at 0.51 ml using a single-syringe infusion pump (SP100iZ; World Precision Instruments Inc., FL, USA). The needle was connected to a 20 kV positive power supply (Gamma High Voltage Research, Inc., Ormond Beach, FL, USA) and directed at a negatively charged, 30-mm-diameter, circular, brass collector at a distance of 25 cm. The drug-loaded, porous, composite scaffold was examined *in vitro* with human dermal fibroblasts and *in vivo* in a mouse model.

### Analysis of scaffold surface morphology by scanning electron microscopy

The surface morphologies of electrospun CP and CPA scaffolds were examined using a JEOL scanning electron microscope (SEM). Specimens were mounted on aluminum sample stubs and sputter-coated with platinum using a JFC-1600 Auto Fine Coater (JEOL DATUM, Tokyo, Japan) prior to examination under the SEM at a voltage of 15 kV.

Samples with attached HDFs were transferred to 10-ml centrifuge tubes and residual culture medium was replaced by sterile PBS (pH 7.4). After rinsing three times with PBS, the HDFs attached to the surface of the materials underwent an initial fixation by addition of 2.5% (w/v) glutaraldehyde in 0.1 M sodium cacodylate buffer trihydrate for 30 min (first fix). Samples were then rinsed with PBS before a second fixation using 2% (w/v) osmium tetroxide in 0.2 M sodium cacodylate buffer for 1 h. Cells attached to the samples were washed with sodium cacodylate buffer trihydrate and dehydrated using a series of ethanol dilutions (50–100%) for 5 min, twice per dilution. Specimens were finally dried in HMDS and prepared and mounted as described above.

### Quantification of pore size and fiber diameter of the scaffolds

Pore size and fiber diameter of CP and CPA scaffolds were quantified under SEM using an established method.[14] The widths of 30 fibers and the diameters of 30 pores randomly chosen from SEM images were measured.

### Cell culture

HDFs were obtained from donated skin biopsies of patients at the Burns Unit in Concord Repatriation General Hospital with approval from the Hospital Human Ethics Committee.

CP and CPA scaffold cells were seeded at 1 × 10^4^ cells/cm^2^ in triplicate. Seeded scaffolds were incubated at 37°C, 5% CO_2_ in DMEM supplemented with 10% (v/v) FBS and 100 U penicillin/0.1 mg streptomycin/0.2 mg neomycin/ml, with the culture medium replaced every 2 days. The cells in the scaffolds were harvested by trypsin digestion and the cell numbers determined by trypan blue staining and viable counting on days 1, 5, 7, 14, and 28 post-seeding.

### Analysis of HDF ingrowth on scaffolds

HDF ingrowths in the CP and CPA scaffolds were analyzed using DAPI staining and fluorescence microscopy. HDF-seeded scaffolds were collected and washed twice with DPBS, followed by a 15-min fixation in 4% (v/v) paraformaldehyde. After fixation, samples were permeabilized in 0.2% (v/v) Triton solution for 15 min. The cell-fixed scaffold was then washed in DPBS three times and air-dried overnight. The nuclei of HDF-seeded scaffolds were stained using ProLong Gold antifade reagent with DAPI and air-dried overnight in the dark. The DAPI-stained HDFs on scaffolds were visualized and photographed using a fluorescence microscope (Axiovert 200; Carl Zeiss, Inc., Jena, Germany)

### Animal model

**Figure 1:**
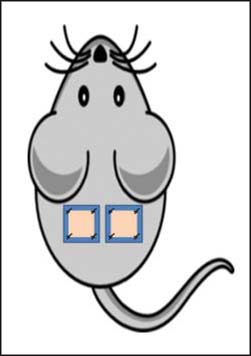
Diagrammatic representation of dorsal open wounds (pink) splinted with silicone frames (outline in blue) and attached to skin with silk sutures (black) at the corners.

A mouse model of wound healing was used to evaluate biocompatibility, efficacy, and cellularization of CP and CPA scaffolds. Specific pathogen-free, female and male BALB/c mice, aged 8 weeks and weighing 21.0 ± 1.9 g (female)/25.0 ± 2.7 g (male) were purchased from the Australian Animal Resources Centre. All animals were acquired, housed, and studied under a protocol approved by the SSWAHS Animal Welfare Committee in Sydney, Australia. Each mouse was individually anesthetized by intraperitoneal injection of a mixture of ketamine (75 mg/ ml) and xylazine (10 mg/ml) at 0.01 ml/g of body weight. The dorsal hair was shaved and the skin cleaned with Betadine solution and then washed with sterile saline. Two 1 × 1 cm^2^ full-thickness wounds were surgically created on the dorsal area with a frame of silicone splint stitched to each wound [Figure 1]. The frame was purposely applied to inhibit the mouse wound healing by contraction and enable the wound closure mainly by cell migration from the surrounding skin.

One wound was grafted with the CP scaffold and the other with the CPA scaffold. Each wound was then covered with IV3000 wound dressings (Smith and Nephew, North Ryde, Australia) for 5 days. After surgery, each mouse was caged individually for the first 2 days and then two mice per cage thereafter with free access to food and water. Carprofen (5 mg/kg) was given at the time of anesthetization and then on the following day post-surgery for analgesia. Wound area was measured using Visitrak Grid (Smith and Nephew) on days 0, 3, 5, 7, 14, and 21 post-surgery Wound area at each time point was then normalized to the original wound area, defined as the wound area at time points divided by the initial wound area ×100. The mice were sacrificed and skin biopsies collected for histological analysis on days 7, 14, and 21 after surgical grafting.

### Histology

Skin biopsies were fixed in 10% formalin for 24 h and the samples from *in vitro* experiments were fixed in 4% paraformaldehyde with DPBS washes before and after fixation. All samples were then dehydrated with ethanol solutions of increasing gradient concentrations and then in xylene. The dehydrated samples were embedded in paraffin wax and sectioned. Sections of 4 μm in thickness were then deparaffinized and stained using Hematoxylin and Eosin.

### Statistical analysis

Data are expressed as mean ± standard deviation. Statistically significant differences were determined by *t*-test and one-way analysis of variance (ANOVA). Statistical significance was accepted at *P* < 0.05 and indicated in the figures as *(*P* < 0.05) and **(*P* < 0.01).

## Results

### Characterization of CP and CPA scaffolds [Figure 2]

During the electrospinning processes, the CPA blend was injected to the negatively charged brass collector to fabricate porous scaffold. The polymer fibers set very quickly. After peeling the scaffold off the collector, no residual drug power or CPA polymer was observed in the electrospinning system, indicating the incorporation of ASC-J9 in the composite fibers.

**Figure 2:**
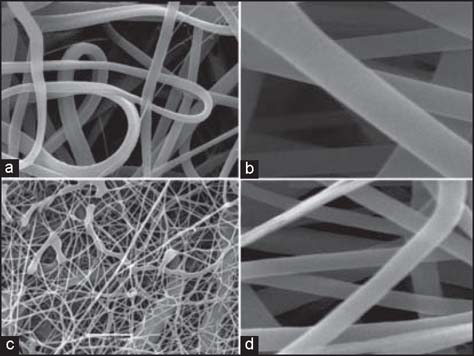
SEM analysis of electrospun collagen (7.5% w/v) and PCL scaffold (7.5% w/v) without (a, b) and with (c, d) ASC-J9 (0.1 mg/ml) incorporated (a and c, ×5000; b and D, ×15,000).

CP scaffolds had a pore diameter range of 1.0–13.6 μm and a fiber diameter range of 0.5–1.8 μm, and CPA scaffolds had a pore diameter range of 0.9–4.7 μm and a remarkable decrease in fiber diameter to the range of 0.1–0.3 μm. The CP fibers were mainly circular and thread-like with occasional flat, ribbon-like fibers, and were uniform except for occasionally thinner fibers, whereas CPA fibers were only circular with a much larger range in fiber size.

It was also observed on SEM examination that CP and CPA fibers had a difference in surface morphology. CP fibers had a uniformly smooth surface, while CPA fibers demonstrated relatively rough and irregular surface.

### Interaction of human dermal fibroblasts with electrospun scaffolds

**Figure 3:**
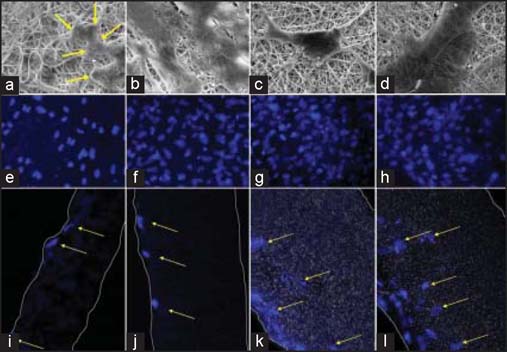
Human dermal fibroblasts in CP and CPA scaffolds by SEM and DAPI staining. Sample images represent, respectively, the HDFs on CP scaffold at day 7 (a, e, i) and day14 (b, f, j), and HDFs on CPA scaffold at day 7 (c, g, k) and day 14 (d, h, l).

SEM analysis and DAPI staining showed the attachment and proliferation of HDFs in both scaffolds [Figure 3]. Subconfluent HDF growth on CP scaffolds was seen by day 14 and HDF ingrowth in CP scaffolds was limited throughout the time course. However, increased attachment and proliferation was seen on CPA scaffolds at days 7 and 14, and significantly more HDFs infiltrated the CPA scaffolds in comparison to CP scaffolds.

**Figure 4:**
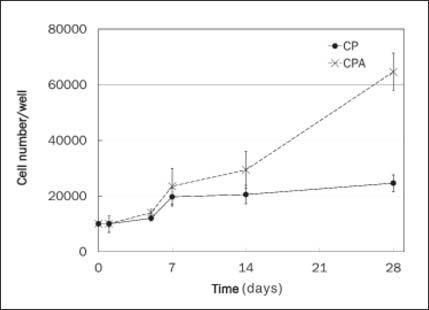
HDF proliferation on CP and CPA scaffolds over 28 days (*n* = 2). The cell proliferation in CPA at day 14, 21, and 28 were significantly higher.

Cell numbers increased over time on both CP and CPA scaffolds [Figure 4]. Cell numbers on CP scaffolds increased twofold at days 7 and 14 post-seeding and about 2.5-fold by 4 weeks post-seeding. Cell numbers on CPA scaffolds increased 2.8-fold between days 1 and 14 post-seeding and by 6-fold over 4 weeks.

### Animal model study of CP and CPA scaffolds

**Figure 5:**
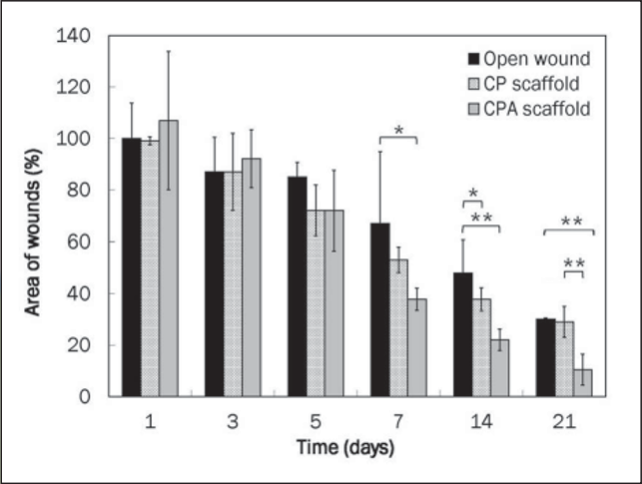
Wound area profile over 21 days of CP- and CPA-grafted wounds. CPA accelerated wound closure in mice (*n* = 3–4, *P < 0.05 and **P < 0.01).

In animal models, electrospun CPA grafts accelerated wound healing, compared to open wounds and wounds grafted with electrospun CP scaffolds [Figure 5]. Raw wound area in CPA-grafted animals was significantly reduced by days 7, 14, and 21, compared to CP-grafted wounds. By day 21, the wounds in CPA-grafted mice were almost fully healed while over 30% of wound areas in both CP-grafted and open wound control groups were still not open.

**Figure 6:**
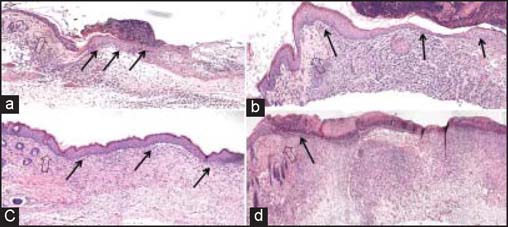
Histological analysis of wound healing following CP and CPA grafting. Accelerated keratinocyte migration and re-epithelialization was seen in CPA-grafted mice. (a) CPA at day 5, (b) CPA at day 7, (c) CPA at day 14, and (d) CP at day 14. Each large arrow points to wound edge, while the small black arrows highlight keratinocyte migration and epidermal development.

In the animal model, the silicone splint inhibited wound healing by contraction. Comparing CPA-grafted mice to CP-grafted mice, CPA scaffolds accelerated keratinocyte migration over the wound edge toward the central area for more effective wound closure and re-epithelialization [Figure 6].

## Discussion

In addition to scaffolds and skin progenitor cells, biofactors, including growth factors, hormones, and inflammatory regulators, and their regulation are critical for wound healing and skin regeneration. While bioscaffolds provide structural support, and guidance for the attachment and migration of dermal fibroblasts and keratinocytes, the biochemical factors and their regulation further induce and promote the cell proliferation, ingrowth, and differentiation for more effective wound healing and regeneration of dermal and epidermal tissues. In cell culture, the defined media provide a rich source of essential nutritious ingredients, biofactors, and other supplements. Under *in vivo* situation, the essential supply needed for wound healing and skin regeneration could only depend on the diffusion of nutritious ingredients, vascularization, and blood supply in the wound region. Fabrication of bioactive factors in the scaffold would provide the cells with extra supplements to ensure more effective cell migration, proliferation, or differentiation in addition to bioscaffolding and, therefore, accelerate tissue regeneration and wound closure.

Electrospun CP and CPA scaffolds were produced and they displayed fibrous, porous structures with fiber diameter ranging from 0.1 to 4.0 μm [Figure 2]. The electrospun fibers were morphologically similar to native extracellular matrix fibers and provided a large surface area to volume ratio, thereby promoting cell interactions and new tissue formation.[15,16] HDFs were shown to attach, migrate, and proliferate on the CP scaffold [Figure 3a, b] with enhanced cell proliferation, compared to PCL only scaffolds (data not shown). This finding suggested that the collagen polymer protruding on the surface of CP scaffolds enhanced surface hydrophobicity and resulted in favorable cell interaction, proliferation, and migration. SEM analysis of seeded fibroblasts at day 14 on CP scaffold revealed large numbers of attached cells in contact with the surface [Figure 3b]. Pronounced flattening and spreading of cells was observed at days 7 and 14 [Figure 3a, b], obscuring the underlying polymer surface, with cellular processes connecting the flattened cell bodies. This behavior suggests strong cell adhesion with the underlying substrate via local contacts and provides a measure of the biocompatibility of the polymer. However, HDF penetration of the CP scaffolds was not observed due to either the small pore size in the range of 1.0-13.6 μm of the scaffolds, uneven distribution of collagen throughout the fibers, or the limited time available for cell ingrowth to occur. These findings are broadly in line with those of Powell *et al.*[17] who found that collagen-PCL solution with more than 30% (w/v) PCL resulted in separation of the collagen and PCL phases within the fibers. The lack of uniformity may slow or impair cell attachment and migration, as the cells have to attach to a natural polymer domain and then a synthetic polymer domain. Furthermore, the harsh processes of CP electrospun fabrication could also reduce the effectiveness of collagen element in the CP composite for cell migration.

In the study, we explored the feasibility of making a bioactive scaffold, not only for drug delivery but also to improve the scaffold’s functionality for better cell growth, migration, and accelerated wound healing. An important aspect of drug delivery is the stability and activity of the drug during formation and throughout the release period. Although electrospun scaffolds were used for controlled delivery of low-molecular-weight hydrophilic drugs, growth factors, and proteins, to retain a lasting activity of delivered factors has always been a challenge due to the harsh processes of fabrication.[17] Compared to other protein factors such as EGF and FGF, the AR inhibitor, ASC-J9, is a chemically stable compound that could better survive the process and retain its activity. Under SEM, the CPA scaffolds displayed uniformly thinner fibers and relatively more porous structures, compared to CP scaffolds [Figure 2]. In comparison with CP fibers, fiber morphology by SEM analysis revealed that CPA fibers were thinner with much rougher fiber surface. The absence of obvious drug particles on the CPA fiber surface suggested that dispersed ASC-J9 was effectively coated by collagen-PCL polymers during fiber formation. The *in vitro* data demonstrated that CPA scaffolds enhanced faster cell proliferation and better cell—matrix interaction over the time course of 28 days, with significantly increased overall cell numbers in the scaffolds post-cell seeding, particularly from day 7 onward. This demonstrated that the drug and scaffold were not cytotoxic to the cells. These findings reflected a highly efficient delivery and sustained release of active ASC-J9 from CPA scaffolds over 4 weeks, which was also evident by DAPI staining that revealed HDFs migrated through to the central region of the CPA scaffolds on day 7 and by day 14 post-cell seeding. The observation of gradually accelerated cell migration and distribution in CPA scaffolds was encouraging, as it proved the feasibility of utilizing CP electrospun scaffolds for incorporation and delivery of bioactive molecules to promote cellular activities including cell—scaffold interaction, cell proliferation, and migration.

In our animal model, CPA scaffolds were superior to CP scaffolds in promoting wound healing. Significantly accelerated migration of epidermal migration and wound closure was associated with CPA-grafted wounds, with only a mild inflammatory response observed, when compared to CP scaffolds. The newly formed skin had a well-differentiated epidermis with multiple layers of keratinocytes and a vascularized neodermis, both important components for functional skin. Our *in vivo* findings together with the above *in vitro* data were in agreement with the previous studies which demonstrated that topical application of ASC-J9 cream on mice resulted in accelerated wound repair via increased fibroblast proliferation and keratinocyte migration following inhibition of AR function.[9] In comparison to topical treatment using cream or gel, CP electrospun scaffolds could serve as a good delivery device for not only structural scaffolding but also sustained delivery of bioactive compounds or molecules with minimal burst release for more effective wound healing and re-epithelialization.

Taken together, accelerated fibroblast proliferation, re-epithelialization, and wound healing were associated with grafting CPA scaffolds. These results were achieved via the sustained release of ASC-J9 from CPA scaffolds into the wound area to promote cell proliferation and migration in addition to the structural support and guidance. More importantly, this study demonstrated the principle of using electrospun CP scaffolds for releasing drugs or active molecules into the wound. It could enable the tissue regenerative scaffold, with extra biofactors or cues, to speed up skin cell growth, migration, wound closure, and re-epithelialization. Future studies will focus on refining the process to improve the fabrication of CP scaffolds for delivering ASC-J9, and possibly multiple biofactors, as well as characterizing host - scaffold interactions including inflammation, cell recruitment, and synthesis of key proteins in relation to wound healing, skin regeneration, and scar development.
